# Cleaning performance of electric toothbrushes around brackets applying different brushing forces: an in-vitro study

**DOI:** 10.1038/s41598-024-56017-1

**Published:** 2024-03-11

**Authors:** Reto L. Rominger, Raphael Patcas, Blend Hamza, Marc Schätzle, Florian J. Wegehaupt, Monika A. Hersberger-Zurfluh

**Affiliations:** 1 Clinic of Orthodontics and Pediatric Dentistry, Center of Dental Medicine, University of Zurich, Plattenstrasse 11, 8032 Zurich, Switzerland; 2 Clinic of Conservative and Preventive Dentistry, Center of Dental Medicine, University of Zurich, Zurich, Switzerland

**Keywords:** Oral diseases, Orthodontics

## Abstract

Throughout treatment with fixed orthodontic appliances, effective plaque control is crucial to maintaining dental health. This in-vitro study evaluated the cleaning performance of eleven different brush heads of seven electric toothbrushes (oscillating-rotating and sonic motions) and varying brushing forces around orthodontic brackets. Six Mini Diamond^®^ Twin brackets were placed on black-stained front teeth. Teeth were coated with white titanium oxide and brushed in a machine six times for one minute with two different brushing forces (1 N and 1.5 N). Eleven different brush heads were evaluated (either oscillating-rotating or sonic movements). The teeth were scanned and planimetrically evaluated after brushing. Three detailed plaque areas (DPAs) were created: proximal (< 1 mm to bracket), mid-tier (1–2 mm to bracket), and distant (> 2 mm to bracket). The proportion of contaminated proximal, mid-tier, and distant surfaces (white regions) in relation to the respective DPA was calculated. Independent of brushing forces, places with a higher distance (> 2 mm) to the orthodontic bracket had the least amount of residual contamination, followed by areas with a minor (1–2 mm) and proximal distance (< 1 mm). In all of the brushes tested and for both estimated brushing forces, the region with the highest residual contamination was the proximal area. The brush heads of the Paro^®^ Sonic toothbrush left the least amount of residual contamination. The cleaning performance of electric toothbrushes around brackets on upper incisors varied across the brushes examined. The proximal area has the most residual contamination. Furthermore, 9 out of 11 toothbrushes cleaned more successfully with 1.5 N than with 1 N brushing force.

## Introduction

Tooth brushing is of utmost importance to keep the oral cavity healthy. Tooth brushing reduces plaque accumulation on the tooth surface^1^ and prevents diseases in the oral cavity such as gum disease and tooth decay^2^. An earlier study showed that already eight hours after the last tooth brushing, it was possible to find around 10^3^–10^4^ bacteria per mm^2^. The number increased 100–1000 times after 24 hours^3^. This concludes that neglecting oral hygiene practice could increase the risk of developing an oral disease significantly.

There is a major change in the bacterial flora of the plaque in the oral cavity after orthodontic fixed appliances are introduced into the oral cavity^4^. Therefore, efficient plaque control is an essential component in the maintenance of dental health during fixed orthodontic appliance treatment^5,6^. Fixed orthodontic appliance treatment is the most common type of orthodontic device. It is an orthodontic appliance with attachments that apply force via arch wires or auxiliaries. The devices are intended to correct dental irregularities. Studies showed that fixed appliances change the subgingival microflora and increase the growth of periodontopathogenic bacteria^7^. Furthermore, due to the altered oral hygiene situation, there is a higher risk of developing white spot lesions, which are apparent as huge decalcified patches with or without cavitation in some patients; in other cases, they appear as tiny lines surrounding the brackets^5,8^. The presence of orthodontic appliances may produce a transitory increase in bacterial concentration and isolation rate of oral streptococci^9^. Fixed appliance components such as brackets, molar bands, and archwires are all hotspots for plaque accumulation, with an increased risk for creating white spot lesions on enamel surfaces^10^. Despite improvements in materials and preventive efforts, orthodontic treatment continues to carry a considerable risk of enamel demineralization^11^. Numerous preventive methods have been recommended to prevent or reduce the development of white spot lesions during fixed appliance treatment, including fluoride-releasing glass ionomer cements for bonding and banding^12^, daily use of a fluoride mouth rinse^13^, the use of lingual orthodontic appliances^14^ and bonding brackets on a caries-protective adhesive patch^15^. Nevertheless, such measures are dependent on either patient compliance or frequent professional oral hygiene. Petrauskiene and his group showed in their study that patients with orthodontic appliances mostly have a better awareness of oral hygiene and take tooth cleaning more seriously^16^.

Plaque removal by toothbrushing is still the most commonly accepted and effective preventive method against dental plaque^17^. Different types of toothbrushes have been designed and promoted for orthodontic patients. Although it is debatable whether manual or electric toothbrushes are superior, a meta-analysis of 51 studies with over 5000 participants found that electric toothbrushes produced significantly higher outcomes in terms of plaque removal^18^. Additionally, powered toothbrushes (either sonic or rotating) may also increase patients' motivation, which is very valuable and helpful over an overall treatment time of two years or more^19^. Powered toothbrushes equipped with high-frequency sonic and oscillating-rotating technologies are currently the most widely available commercial devices worldwide. Sonic toothbrushes generally vibrate at 24,000–40,000 strokes per minute. In comparison, oscillating electric toothbrushes rotate at around 1300–8800 strokes per minute^20^.

The purpose of this study was to evaluate the cleaning performance of eleven different brush heads from seven different electric toothbrushes currently on the market in Europe under standardized laboratory conditions using a well-established test method^21,22^ with varying brushing forces, as well as to measure enamel areas with inadequate filament contact in a custom-made model of an upper anterior segment with bonded brackets.

## Materials and methods

Seven electric toothbrushes (Candida Power (Migros-Genossenschafts-Bund, Zurich, Switzerland), Curaprox Hydrosonic Ortho (Curaden AG, Kriens, Switzerland), Oral-B^®^ Genius, Oral-B^®^ Pulsonic Slim (P&G corp., Cincinnati, USA), Paro^®^ Sonic (Esro AG, Kilchberg, Switzerland), Philips Sonicare Diamond Clean (Philips AG, Amsterdam, Netherland), Waterpik^®^ Sensonic^®^ Professional Plus (Water Pik Inc., Fort Collins, USA) of two different modalities (oscillating-rotating and sonic movements) with a total of eleven different brush-heads were tested (Table 1 and Supplementary File 1).

**Table 1:** Technical data and characteristics for different toothbrushes and brush heads tested in the present study.

	Manufacturer	Toothbrush	Brushhead	Features	Used modus	Bristle typ by manufacturer	Oscillation by manufacturer (movements per minute)	Measured bristles displacement	Modality
A	Candida	Power	(a) Power	2 min timer, 3 cleaning-modes, 30 s. interval-timer	1 (high)	n/s	40′000	5 mm	Sonic
B	Curaprox	Hydrosonic ortho	(b) Power (c) Sensitive	7 cleaning-modes, 30 s. interval-Timer, Interdentalbrushhead, Travel-case,	7 (smile)	(b) Medium (c) Extra soft	42′000	(b) 7 mm (c) 7 mm	Sonic
C	Oral-B®	Genius 10100S	(d) Orthocare essentials (e) Sensi ultrathin	2-min timer, 6 cleaning-modes, 30 s. interval-timer, Travel-case, Smartphone holder, 2- pin refill holder,	1 (daily clean)	n/s	–	–	Rotating oscillating
D	Oral-B®	Pulsonic slim	(f) Pulsonic	2-min timer, 3 cleaning-modes, 30 s. interval-timer, extra small design,	1 (daily clean)	n/s	31′000	1 mm	Sonic
E	Paro®	Sonic	(g) Duo-clean (h) Soft-clean	2 min timer, 3 cleaning-modes, 30 s. interval-timer, Interdentalbrushhead,	1 (high)	(d) Soft (e) Extra soft	40′000	(g) 3 mm (h) 3 mm	Sonic
F	Philips Sonicare	Diamond clean amethyst (HX 9370)	(i) Pro result big (j) Pro result small	2-min timer, 5 Cleaning-modes, 30 s. interval-timer, Travel-case,	1 (clean)	(f) Medium (g) Medium	31′000	(i) 3 mm (j) 3 mm	Sonic
G	Waterpik®	Sensonic® professional plus (SR-3000E)	(k) Small	2-min timer, 2 cleaning-modes, 30 s. interval-timer, Travel-case, Interdentalbrushhead,	2 (power)	n/s	30′500	4 mm	Sonic

**Figure 1:**
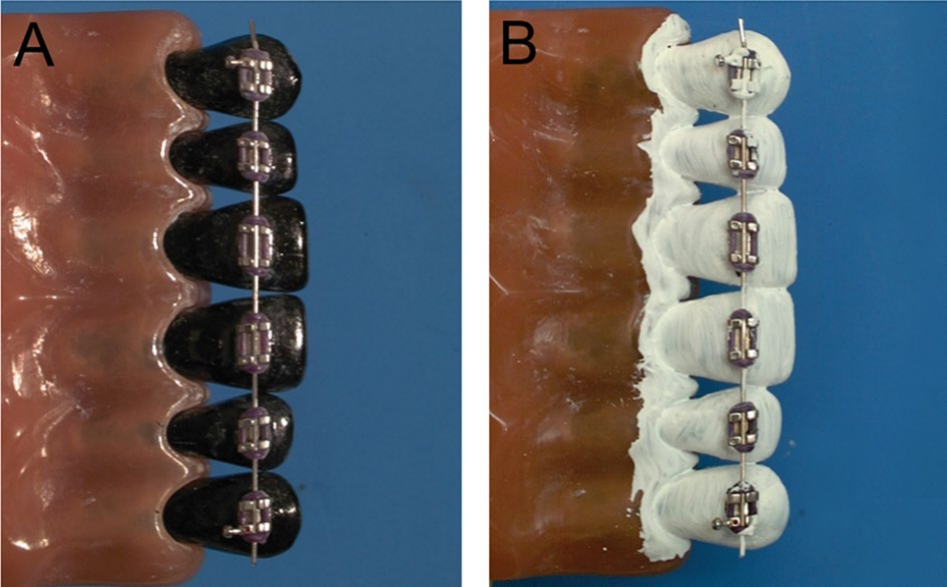
Custom-made tooth model of an anterior segment with brackets bonded to the labial surfaces (Mini-Diamond™ brackets). (**A**) The original black models correlate to 0% residual contamination. (**B**) Colored in titanium oxide correlating to 100% residual contamination.

The toothbrushes were mounted on an automatic brushing machine, which moved the brush heads over a specifically made model, which consisted of six anterior teeth (13–23) made from black plastic and showed mild gingival recessions. On the labial side of the teeth, Mini Diamond^®^ Twin brackets (Ormco BV, Amersfoort, Netherlands) were placed with Transbond™ XT (3 M Unitek, Monrovia, California, USA) according to the manufacturer’s guidelines. The brackets were connected with a 0.016 × 0.022-inch stainless steel archwire (Ormco BV, Amersfoort, Netherlands) (Fig. 1A). This size of wire is typically used in orthodontic therapy after teeth have been leveled for the majority of the treatment period in an 18-slot system.

Prior to the studies, the black model teeth were white coated with a 1:2 solution of titanium oxide in 26 vol percent ethanol, imitating 100% plaque deposition on the tooth surfaces (Fig. 1B). This powdery covering cannot be peeled off extensively, however, it is removed selectively from filament-touched areas. Tooth surfaces that became black after being contacted by toothbrush filaments were considered possibly cleansed, and those that remained white were considered still contaminated.

**Figure 2:**
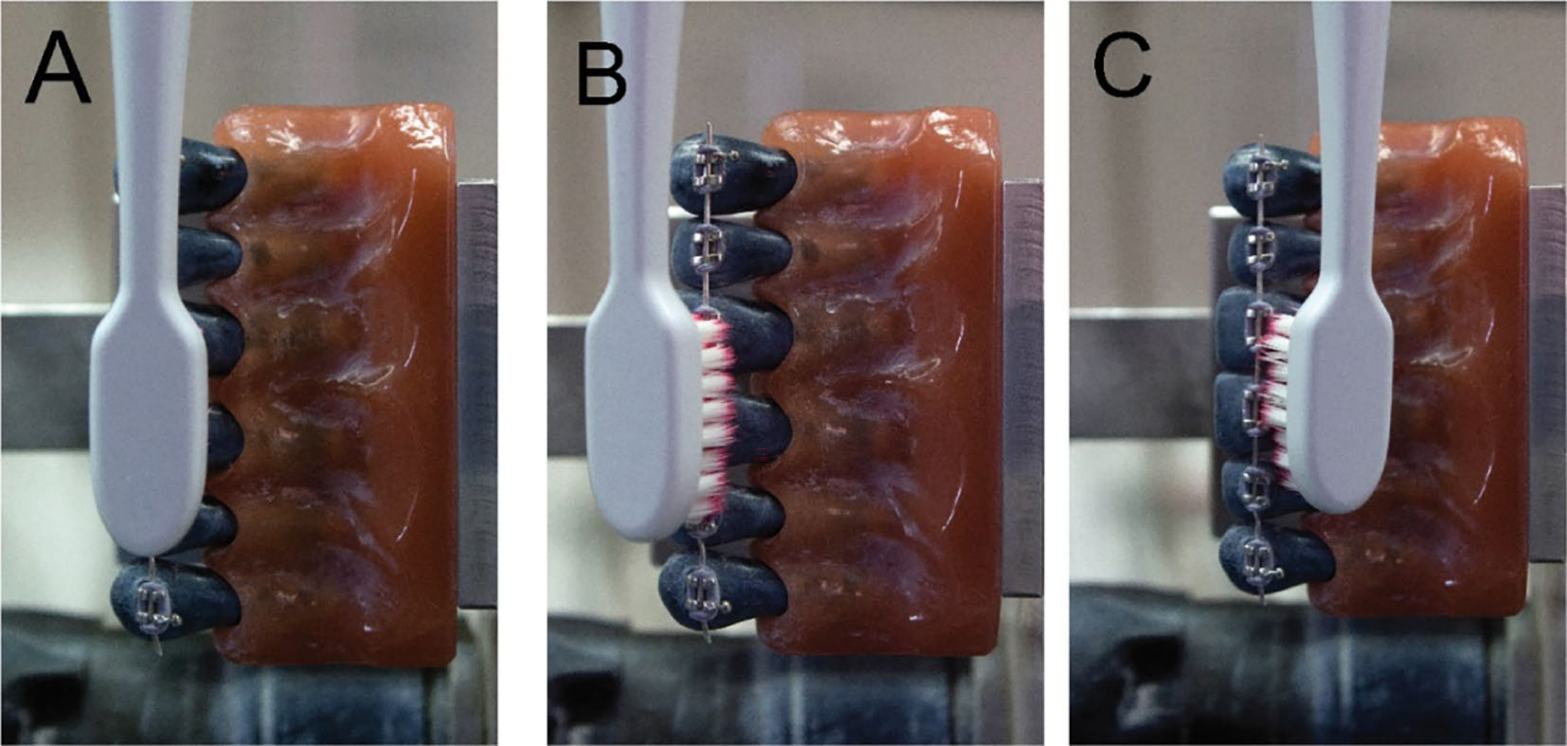
The three cleaning procedures: (**A**) perpendicular cleaning procedure. (**B**) 45° incisal cleaning procedure. (**C**) 45° cervical cleaning procedure.

According to a related investigation^23^, there was little filament-tooth surface contact and ineffective cleaning when the brush heads were positioned perpendicular to the brackets' centres. The performance was greatly increased by tilting the brush head incisally or cervically 45 degrees in the direction of the brackets. As a result, the cleaning process included three steps: The first procedure was perpendicular to the wire, replicating the common *scrub method*, followed by two procedures, each 45 degrees bent incisally and cervically (Fig. 2A–C, respectively). Each procedure lasted for 20 s and cleaned in five steps from tooth 13 to tooth 23 or reversed. The canines were used to turn the toothbrush and were not considered in this examination. At the end, there was a total cleaning time of one minute, which corresponded to ten seconds per tooth. In this investigation, two distinct contact forces (1 N, 1.5 N) were adjusted using a spring balance. If a toothbrush had multiple speed levels, cleaning was performed only at maximum speed.

**Figure 3:**
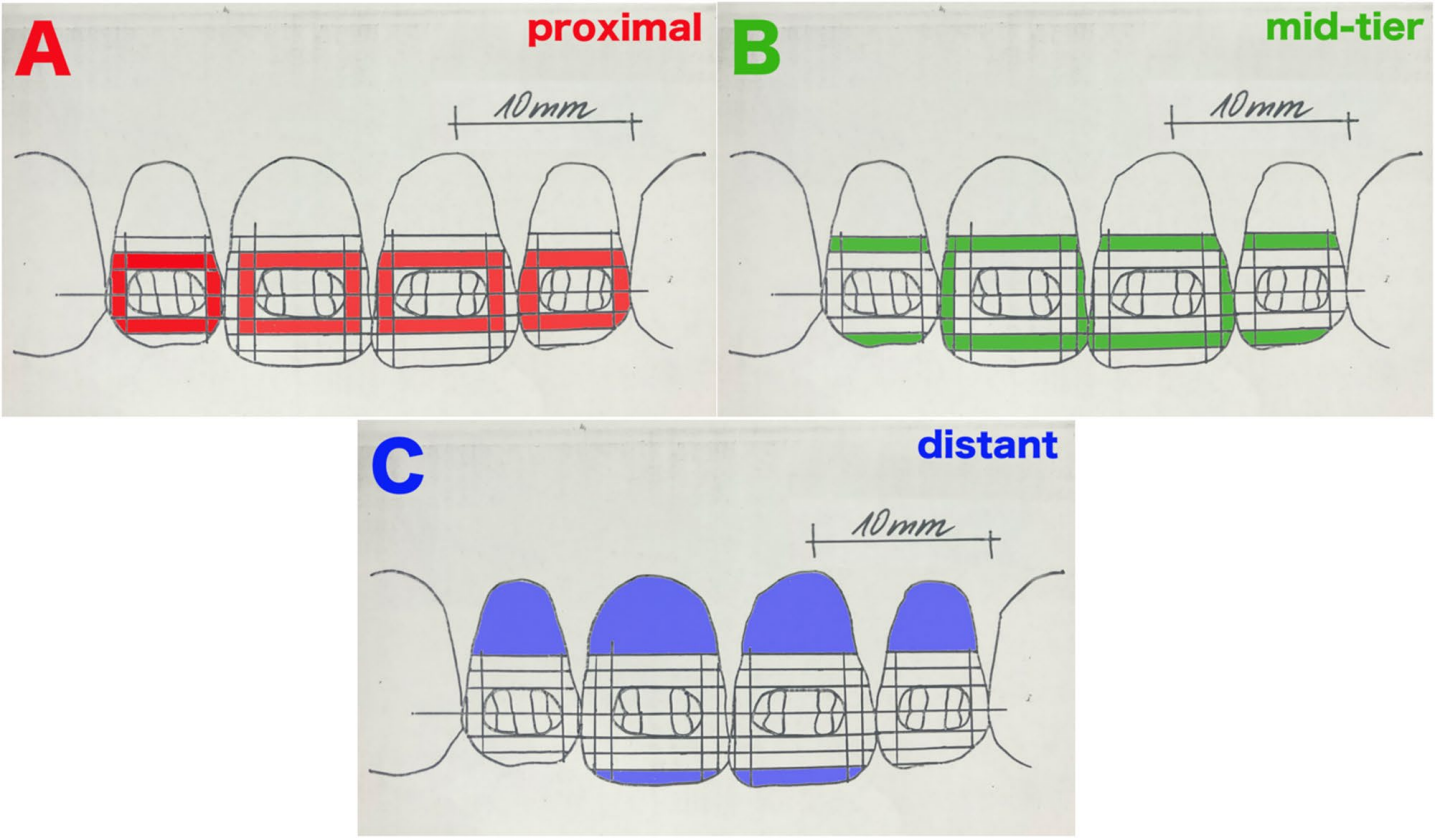
Picture of the three DPAs – detailed-plaque-area. (**A**) Proximal: area proximal to orthodontic bracket (< 1 mm). (**B**) Mid-tier: area with minor distance to orthodontic bracket (1–2 mm). (**A**) Distant: area with larger distance to orthodontic bracket (> 2 mm).

After the cleaning process was completed, the model was scanned with the help of a modified scanner (Hewlett-Packard, Development Company, Palo Alto, USA). The contaminated areas were transferred individually to an identically designed teeth model on transparent film by coloring the analogue area using a mask. Three detailed plaque areas (DPAs) were defined on each tooth: proximal (area proximal to orthodontic bracket (< 1 mm)), mid-tier (area with minor distance to orthodontic bracket (1–2 mm)), and distant (area with greater distance to orthodontic bracket (> 2 mm)). This also ensured the superimposition of the mask on the scanned tooth (Fig. 3A–C, respectively). Areas that did not lose the white coating material (remained uncleaned) were planimetrically quantified with a custom-made software. Using the same software, the proportion of contaminated proximal, mid-tier, and distant surface (white areas) in relation to the respective DPA was calculated^24^. The mean total area of the brackets (63.44 mm^2^) was subtracted from the mean total area of teeth of 321.20 mm^2^ (tooth 12:65.27 mm^2^; tooth 11:97.04 mm^2^; tooth 21:94.81 mm^2^; tooth 22:64.08 mm^2^) resulting in 257.76 mm^2^ as a total of 100 per cent tooth surface. To eliminate bias, the cleaning process was repeated six times with each brush head. The mean of these six values was then computed. The abovementioned scanning of the teeth and the quantification of the residual contamination were carried out by a different operator, who was blinded to the groups/toothbrushes.

In addition to this measurement, we also calculated the variance of the toothbrushes tested over all measurements. The aim here was not to examine how well a toothbrush performs on average but to determine if it regularly provided this value. A significant variance is thus undesirable and suggests that the toothbrush cleans better at times than at others and that the toothbrush is less trustworthy. The reason for this calculation was that a toothbrush must not only clean well, but it must also clean consistently well.

A toothbrush profile with technical information and characteristics for each toothbrush was created. The bristle displacement was measured by flashing a strobe light on the bristles while the toothbrush was positioned on a scale and the bristles moved in the specific mode.

### Statistical analysis

SPSS was used to examine the data (IBM SPSS Statistics for Windows, version 24 [IBM Armonk, NY, USA]). The percentage of contaminated tooth surface in proportion to the respective detailed plaque area (DPA) was investigated descriptively for all tested brushes at each applied brushing force.

**Figure 4:**
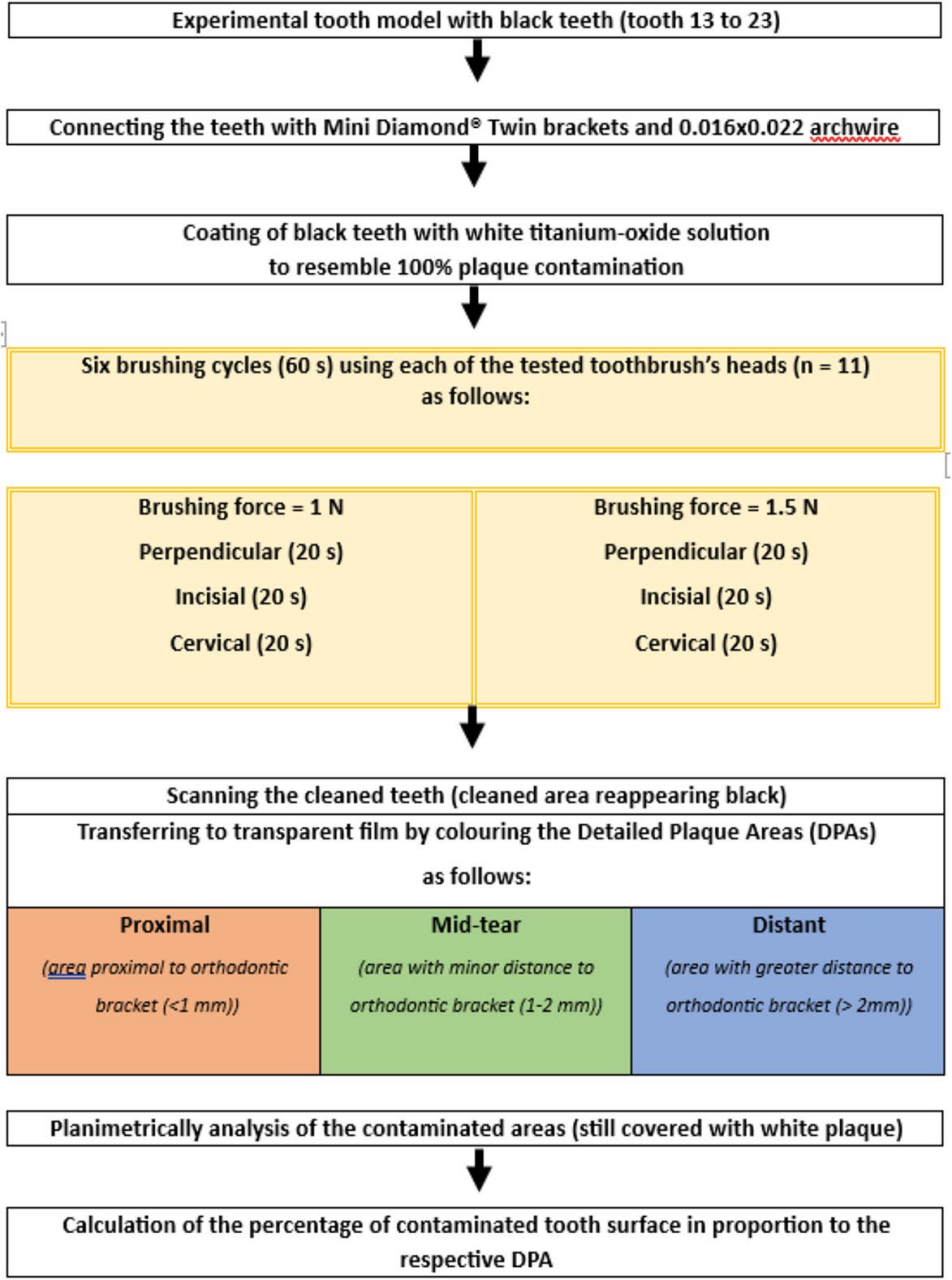
Flow chart of the study.

Because of the high number of different toothbrush types evaluated, it was decided to avoid hypothesis-driven statistics. Performing statistical testing on 11 groups (i.e. 11 toothbrush types) would generate 55 individual evaluations, which have to be further adjusted for direct comparison. This ultimately leads to mostly insignificant differences, even in the case of large variations in cleaning performances. Figure 4 depicts a flowchart of the study design.

## Results

**Figure 5:**
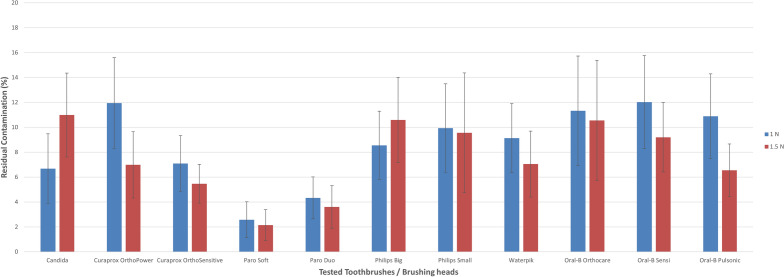
Planimetric assessment of the total residual contamination of all tested brush heads for proximal regions using 100-g and 150-g brushing forces.

**Figure 6:**
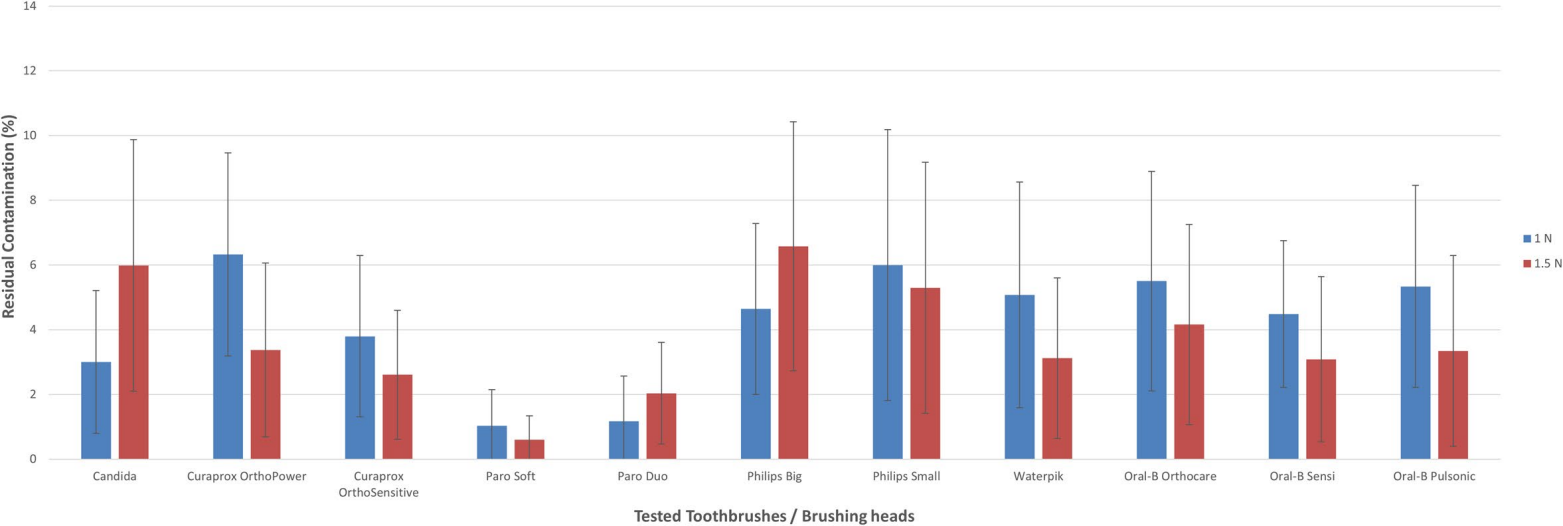
Planimetric assessment of the total residual contamination of all tested brush heads for mid-tier regions using 100-g and 150-g brushing forces.

**Figure 7:**
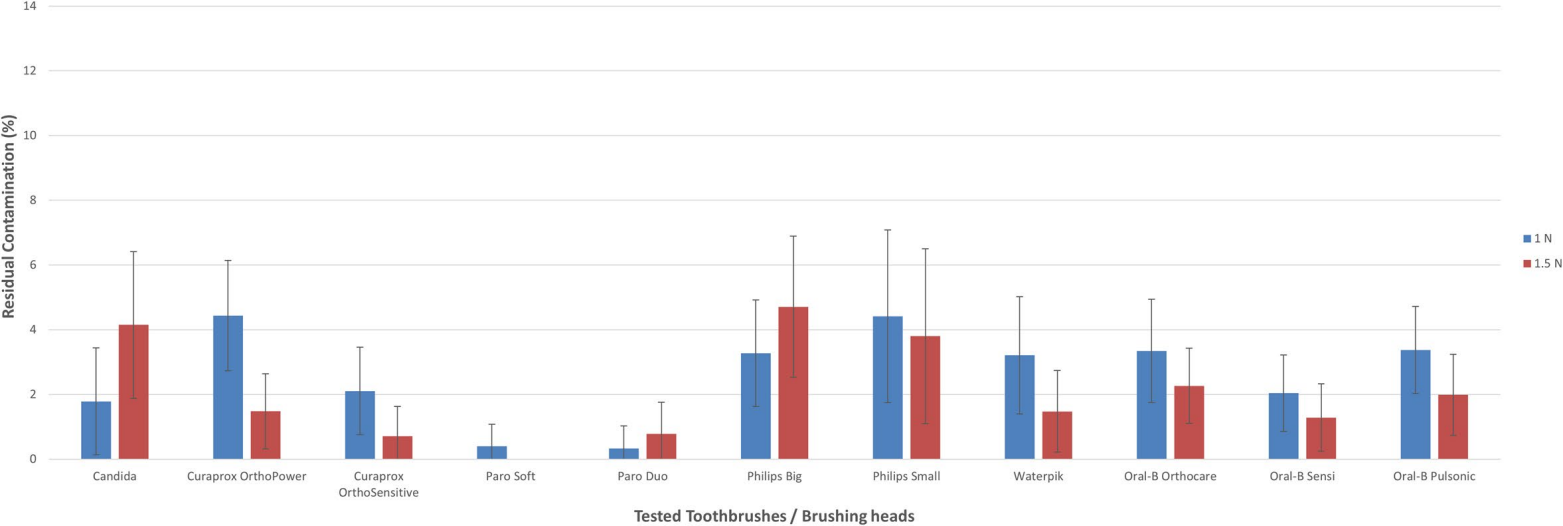
Planimetric assessment of the total residual contamination of all tested brush heads for distant regions using 100-g and 150-g brushing forces.

Figures 5, 6 and 7 illustrate the findings of the planimetric evaluation of the total residual contamination of all tested brush heads at 1 N and 1.5 N brushing forces.

Although most of the toothbrushes (9 out of 11) cleaned better with 1.5 N than with 1 N in proximal regions, only two brush heads (Candida Power and Philips big) cleaned better with 1 N than with 1.5 N. For mid-tier and distant regions, the similar brush heads in combination with the Paro^®^ duo-clean brush head worked better with 1 N brushing force.

For 1 N brushing force, the soft-clean brush-head of the Paro^®^ Sonic toothbrush showed the lowest residual contamination not only in the proximal (2.6%, SD: 1.4) but also mid-tier regions (1.0%, SD 1.1), followed by the duo-clean brush-head, which performed best in areas with a greater distance to orthodontic bracket (> 2 mm) (distant: 0.3%, SD: 0.7). However, the Oral-B® Genius (sensi ultrathin: 12.0%, SD: 3.7) and the Curaprox toothbrush (Hydrosonic ortho power) showed the highest residual contamination (11.9%, SD: 3.6) and hence the lowest outcomes in proximal regions (< 1 mm). Again, in regions bigger than 1 mm (1–2 mm), the Curaprox Hydrosonic Ortho power demonstrated the most residual contamination (mid-tier: 6.3%, SD 3.1, distant: 4.4 percent, SD 1.7) and so scored last in both categories.

For 150-g brushing force, the soft-clean brush head of the Paro® Sonic toothbrush showed the lowest residual contamination (distant: 0.0%, SD: 0.0), followed by the brush head of the Curaprox Hydrosonic Ortho Sensitive (0.7%, SD 0.9). However, the Philips big (mid-tier: 6.6%, SD 3.8, distant: 4.7%, SD 2.2) showed the highest residual contamination and hence the lowest outcomes at minor and larger distances to the orthodontic brackets. Once more, the two brush heads of the Paro® Sonic toothbrush demonstrated the best results not only proximal (soft-clean: 2.1%, SD 1.2, duo-clean: 3.6%, SD 1.7) to the brackets but also in areas with minor distance to the brackets (mid-tier: soft-clean: 0.6%, SD 0.7, duo-clean: 2.0%, SD: 1.6).

Of all the assessed brush heads, the least amount of residual contamination was in places with a greater distance (> 2 mm) to the orthodontic bracket, followed by areas with a minor distance (1–2 mm). The Paro^®^ Sonic soft-clean toothbrush left no visible residual contamination in places further away from the bracket. The region with the most residual contamination was the proximal area in all brushes examined and for both assessed brushing forces.

Table 2 displays the toothbrush's residual contamination variance across all measurements. The toothbrushes with the lowest variation over all metrics were the Paro^®^ Sonic brush heads (soft clean: 1.82, duo clean: 2.96). The Curaprox Hydrosonic Ortho sensitive toothbrush took third place (4.33). In comparison, the second brush head of the previously stated toothbrush (Curaprox Hydrosonic power: 16.26), the Philips Sonicare Diamond Clean Small (17.52), and the Oral-B^®^ Genius (orthocare essentials) (20.97) scored poorly.

**Table 2:** Variance of the toothbrushes tested over all measurements.

Toothbrush	Variance over all measurements	Ranking
Candida power	14.17	8
Curaprox Hydrosonic ortho (power)	16.26	9
Curaprox Hydrosonic ortho (sensitive)	4.33	3
Oral-B® Genius (orthocare essentials)	20.97	11
Oral-B® Genius (sensi ultrathin)	12.66	7
Oral-B® Pulsonic	12.65	6
Paro® Sonic (duo clean)	2.96	2
Paro® Sonic (soft clean)	1.82	1
Philips Sonicare diamond clean (big)	10.47	5
Philips Sonicare diamond clean (small)	17.52	10
Waterpik® Sensonic® professional plus (small)	8.30	4

## Discussion

Cleaning the teeth while undergoing orthodontic treatment might be difficult and time-consuming. Previous research has shown that fixed appliances, such as brackets, band springs, and arch wires, limit access to the tooth surface and complicate the mechanical cleaning task to be performed by the patient^25^. Biofilm accumulation on cervical tooth surfaces can cause chronic gingivitis, tissue overgrowth, and enamel decalcification if dental plaque is not sufficiently managed by brushing^26^.

The goal of this study was to evaluate the cleaning performance of eleven different brush heads from two powered toothbrush modalities (oscillating-rotating and sonic movements) in a custom-made model of an upper anterior segment with bonded brackets under standardized laboratory conditions using a well-established test method^21,22^ with varying brushing forces, as well as quantify tooth surface areas with inadequate filament contact. By constructing three DPAs, this study collected data on how well each toothbrush cleans around orthodontic brackets.

Two different brushing forces were applied in this study (1 N and 1.5 N). Wiegand et al.^27^ reported the mean brushing force applied with an electric toothbrush to be around 1 N (0.9 ± 0.1 N). Brushing at 1-N force was also reported to exhibit less abrasive damage to tooth structure compared to higher brushing forces^28^. At the same time, van der Weijden et al.^29^ reported that a regular lower brushing force (1.5 N) was more efficacious in removing plaque than a higher brushing force (3.5 N) using an electric toothbrush. Furthermore, another study concluded that increasing the brushing force over 1.5 N did not result in an improvement in the cleaning efficacy of an electric toothbrush^30^. In the present study, a premise was assumed that a low increase of the brushing force (from 1 to 1.5 N) might aid the filaments of the toothbrushes to better go around the orthodontic brackets and the arch wire and clean the tooth surface.

Independent of the brushing force, the Paro^®^ Sonic toothbrush handle in combination with the Duo Clean and Soft Clean brush heads performed better than all others in combination with their corresponding powered handles. Cleaning the proximal region (< 1 mm) proved to be quite difficult. For all brush heads tested and for both applied brushing forces, the region with the highest residual contamination was the proximal area. The greater the tooth surface area covered with brackets and the more complex appliance components used, the harder it gets for patients to appropriately clean their teeth^5,6^. In this critical area, the Paro® Sonic toothbrush heads outperformed all other brush heads tested. Plaque in this area was reduced less consistently by all other powered brushes.

Except for the proximal area, only three brush heads cleaned better with 1 N than with 1.5 N brushing force (Candida power, Paro^®^ Sonic duo-clean, and Philips big). One of those brush heads has a decreased second layer of soft bristles with round ends in the center of the brush-head (Paro^®^ Sonic duo-clean, Supplementary File 1e,g). These findings are on par with an in vitro study that demonstrated that various toothbrushes clean differently when applied with varied brushing forces^31^. High forces can cause soft or fine bristles to twist, which reduces cleaning effectiveness. Since soft bristles allow penetration into the interproximal and interbracket regions, interaction with the tooth surfaces increases with little force^32^. If just the brushing force is considered, it may be concluded that toothbrushes with soft bristles, such as the Paro^®^ Sonic duo-clean, offer superior cleaning performance with less brushing force, especially for distant and mid-tier areas.

Specially designed manual and electric toothbrush heads have been introduced in an attempt to facilitate plaque management in orthodontic patients. Experiments with hand toothbrushes have demonstrated that staged and V-shaped brush head designs outperform planar brushes in cleaning effectiveness of teeth with fixed orthodontic attachments^22,32,33^. Their therapeutic usefulness in decreasing gingivitis is, however, questionable^34^.

The Braun Oral-B^®^ Genius orthocare brush head, designed for orthodontic patients, showed high residual contamination for both brushing forces (proximal area) and hence a low cleaning performance when using the simulated cleaning approach. Despite this unsatisfactory experimental performance, subsequent research found that the Braun Oral-B® Ortho brush head was as successful as a manual toothbrush at cleaning around fixed orthodontic equipment in clinical testing. In contrast, a recent clinical trial found that using an Oral-B^®^ oscillating-rotating electric toothbrush was well-received by orthodontic patients and their caregivers and resulted in clinically significant plaque reductions in this risk group. Furthermore, most of the patients who participated reported increased enthusiasm to brush their teeth^35^. Participants who used a powered toothbrush with an orthodontic head also had substantial decreases in interdental bleeding^36^.

The most common brushing technique is simulated scrub, which is characterized by uncontrolled horizontal movements parallel to the occlusal plane. It is mostly utilized by children, who have lower manual skills than adults^37,38^. Several studies that compared the plaque-removing effectiveness of different toothbrushing procedures discovered little to no difference^39^. Efficient oral hygiene may be more dependent on the respective user's performance while using any of the standard procedures than on brushing methods^40^.

Several research comparing manual and electric toothbrushes in fixed orthodontic appliance therapy patients found no difference in gingival, bleeding on probing, or plaque indices^36,41–43^. Similarly, there was no difference in plaque removal efficacy or gingival inflammation reduction between electric 3D and manual toothbrushes in a recent randomized controlled trial of adolescents with fixed orthodontic appliances^44^. The authors concluded that regardless of the brush used, orthodontists should focus on improving their patients' dental awareness and oral hygiene, as well as professional prophylaxis and other oral hygiene aids. A systematic review and meta-analysis of the relative effect on plaque index among pediatric patients using electric versus manual toothbrushes, on the other hand, provide strong clinical evidence for recommending electric toothbrushing to pediatric patients as well as those undergoing orthodontic therapy^45^. Another study found that while the tested sonic toothbrush did not outperform a manual toothbrush in decreasing gingival inflammation in adolescent orthodontic patients, plaque scores on the buccal surfaces of teeth with orthodontic brackets were lower. Furthermore, the counts of Streptococcus mutans were much lower in the electric and ultrasonic groups, which should be associated with a lower risk of oral disease^46^.

The current study's principal limitation is its in-vitro design. A comparable in-vitro brushing setting to the one utilized in this investigation was previously described and clinically verified. Lang and co-workers compared the clinical cleaning performance of two manual toothbrushes to the identical toothbrushes' in-vitro cleaning efficacy (artificial teeth model; teeth covered with artificial plaque staining; inside a brushing machine that conducted horizontal, vertical, and rotary movements)^47^. They concluded that the tested robotic toothbrushing may be suggested for repeatable evaluations of the plaque control and cleaning performance of various toothbrush designs and brushing activities. Nonetheless, brushing is a complicated procedure that cannot be simplified to simple brushing strokes. Other aspects (for example, the formation of a slurry between saliva and toothpaste, different brushing pressures and procedures than those studied here, abrasives, and chemical compounds in the toothpaste) should be addressed as well. As a result, the rankings provided in this study for various toothbrushes based on their attained cleaning power should be taken as a general guideline rather than the final assessment of their clinical performance.

Based on the results of this study and within its limitations, it could be concluded that most of the tested toothbrushes (9 out of 11) cleaned more effectively with 1.5 N than with 1 N especially in proximal areas. The Paro® Sonic toothbrush's soft-clean and duo-clean brush heads (extra-soft and soft bristles) left the least amount of residual contamination on a tooth model with fixed orthodontic attachments, irrespective of the brushing force and DPA (proximal, mid-tier, distant). The most residual contamination was found in the proximal region, which was adjacent to the bracket. Consequently, it is possible to assume that minor residual contamination in this location may result in a lower likelihood of enamel demineralization. Although the results need to be confirmed in a clinical investigation, the definition of three DPAs around the brackets appears to be feasible and valuable for comparing the residual contamination of electric toothbrushes around orthodontic brackets.

### Supplementary Information

Supplementary Information.

## Data Availability

The datasets generated during and/or analysed during the current study are available from the corresponding author on request.
