# Supplementary figures and images for: Cleaning performance of electric toothbrushes around brackets applying different brushing forces: an in-vitro study
Source: Sci Rep. 2024 Mar 11;14:5921. doi: 10.1038/s41598-024-56017-1 (PMC10928086; doi:10.1038/s41598-024-56017-1)

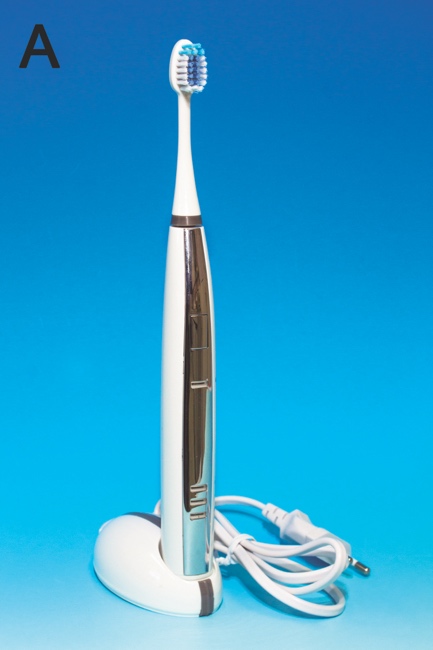

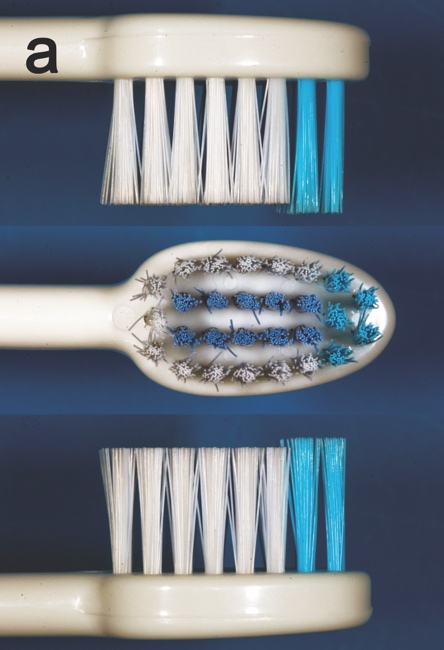


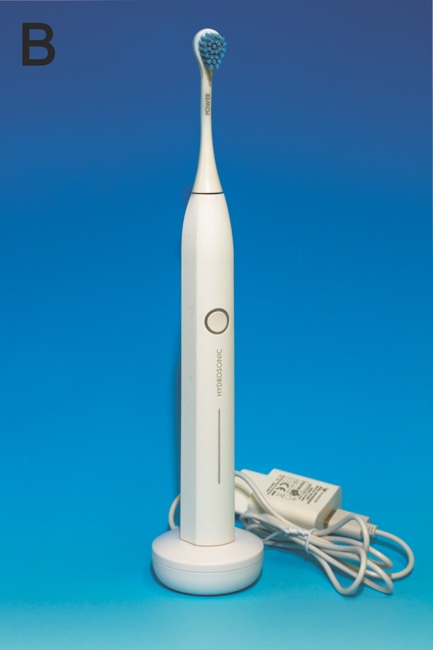

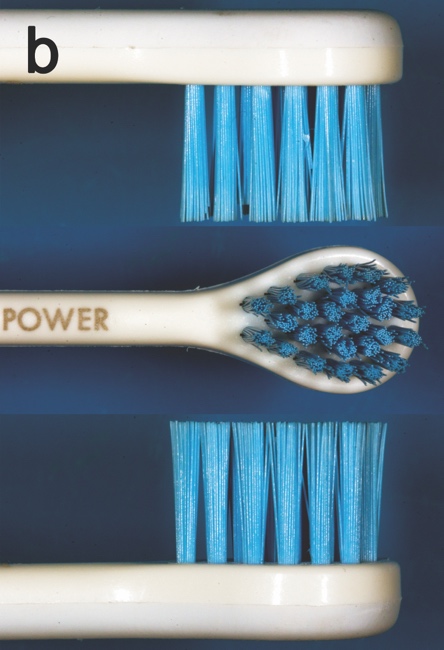

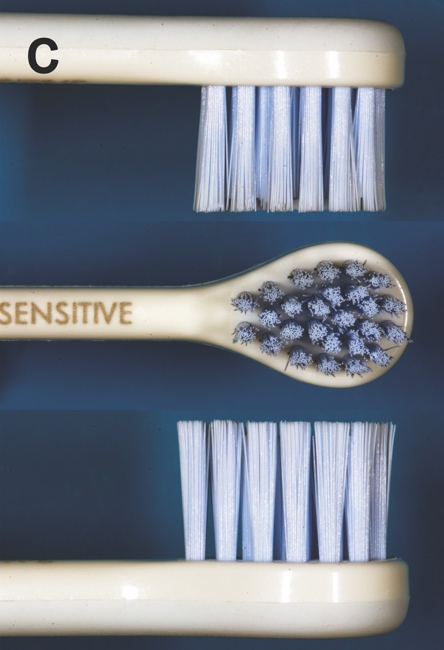


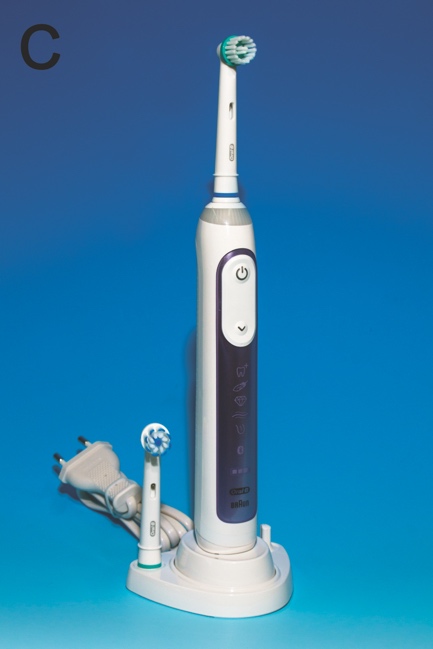

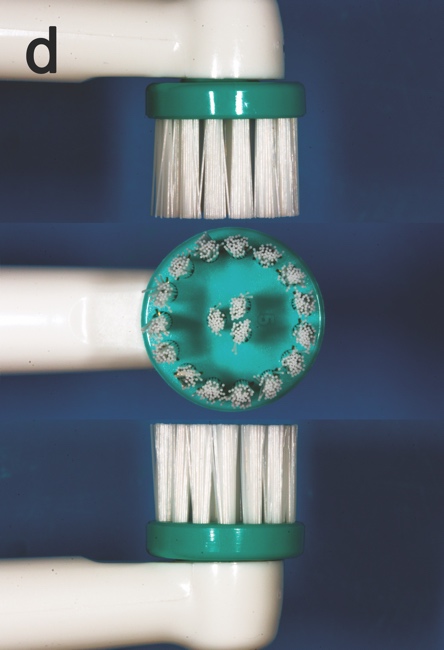

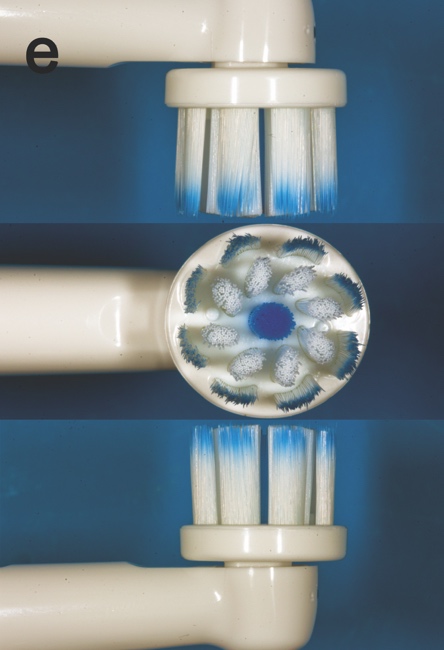


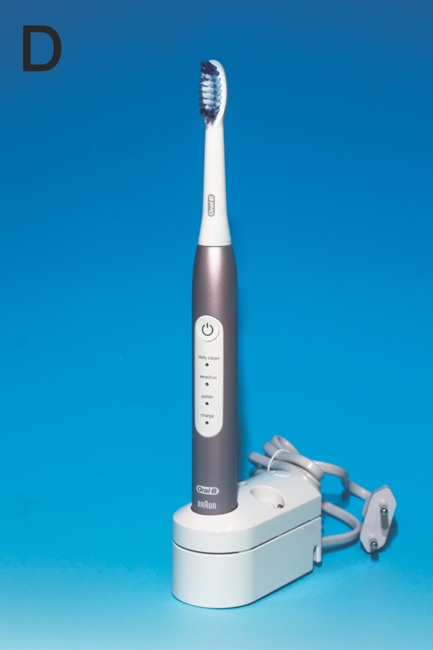

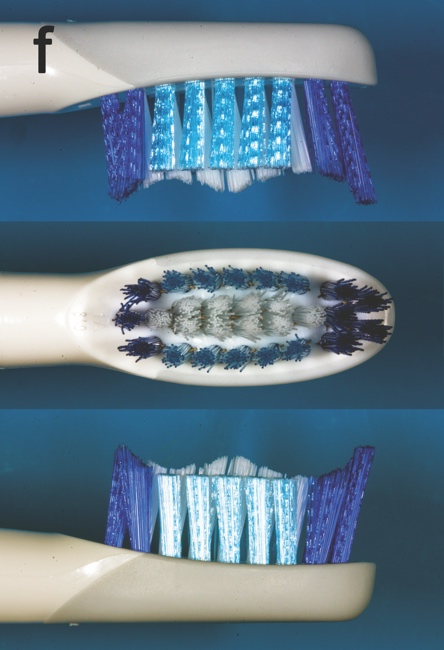


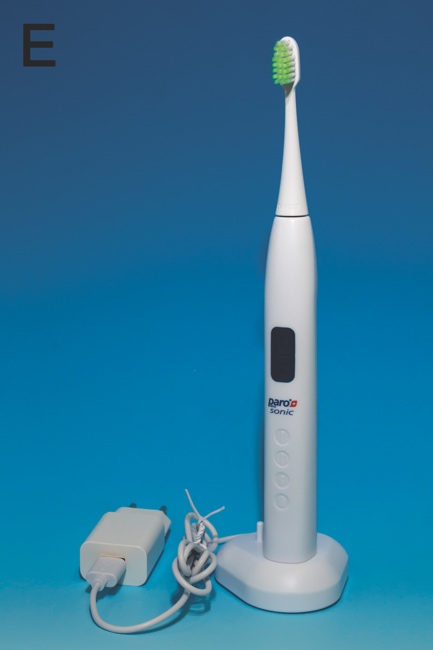

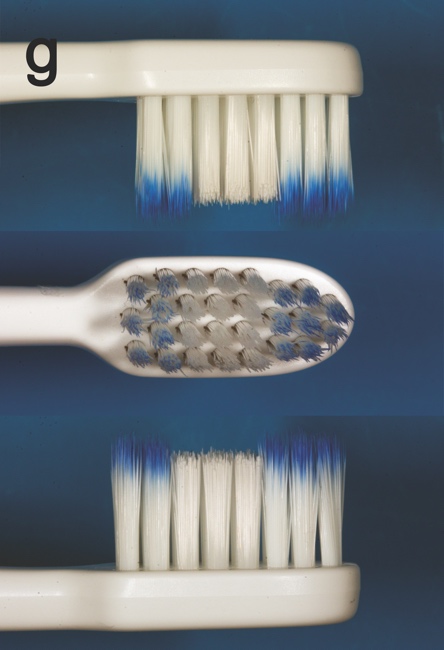

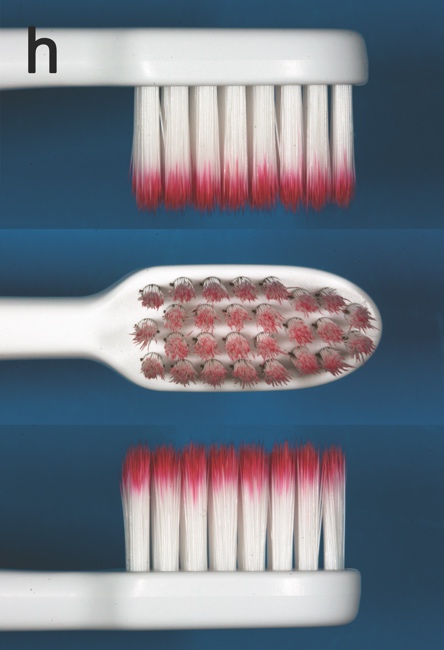


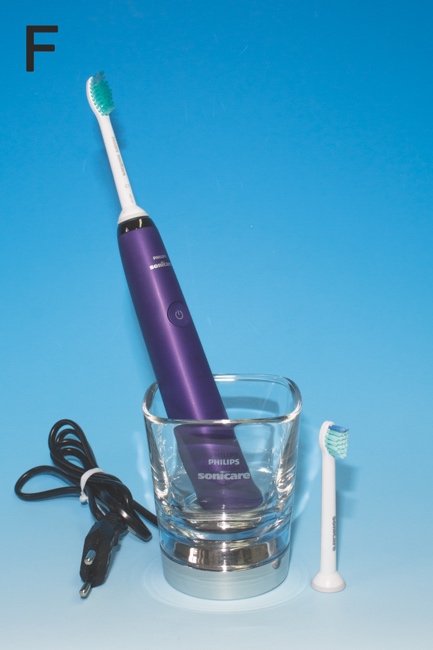

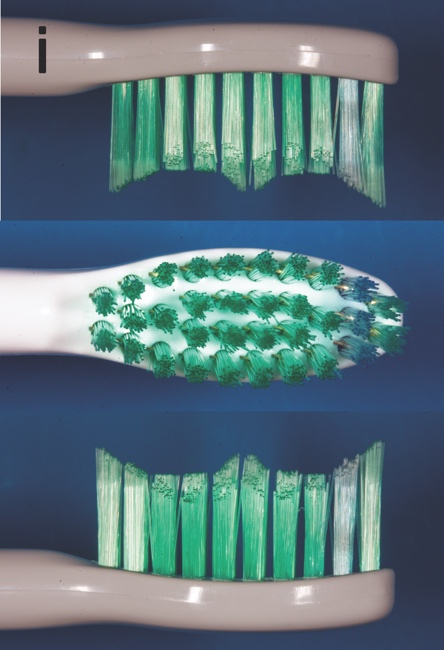

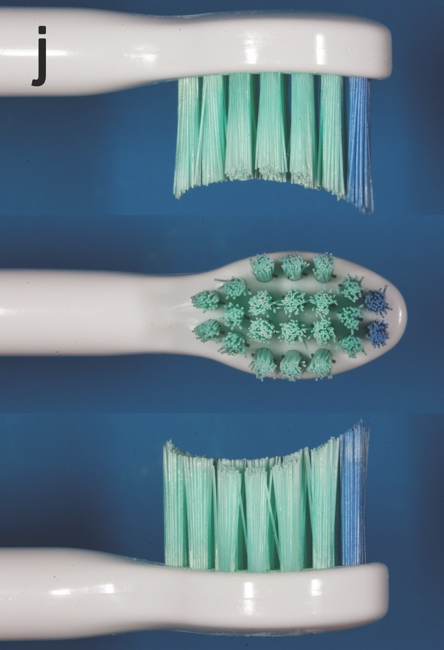


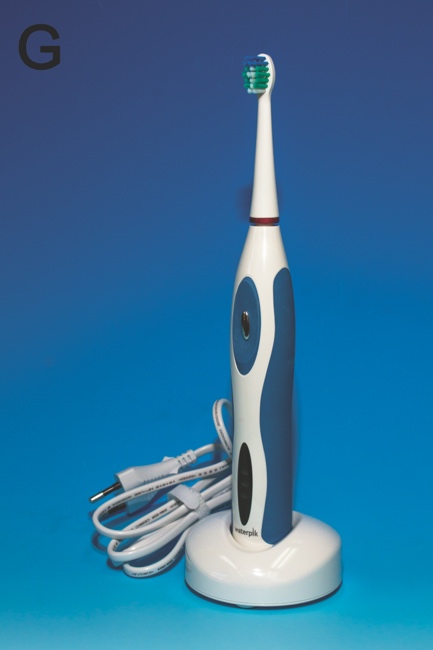

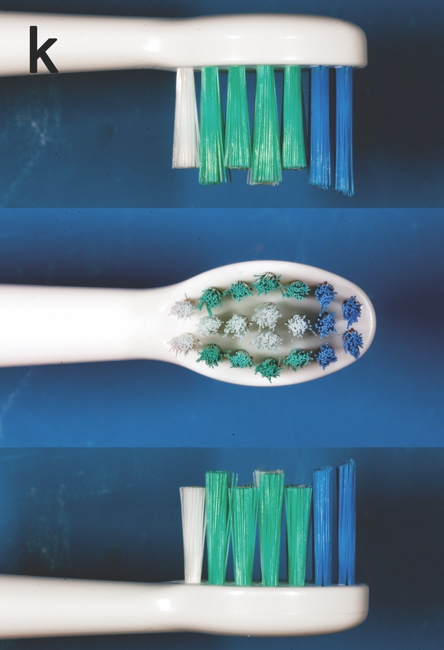


**Supplementary file 1:**Illustration of the eleven toothbrush heads tested

Supplement: Supplementary file 1 — Supplementary Information. [file 41598_2024_56017_MOESM1_ESM.docx]
